# Shaping 3D Path of Electromagnetic Waves Using Gradient‐Refractive‐Index Metamaterials

**DOI:** 10.1002/advs.201600022

**Published:** 2016-03-15

**Authors:** Wei Xiang Jiang, Shuo Ge, Tiancheng Han, Shuang Zhang, Muhammad Qasim Mehmood, Cheng‐Wei Qiu, Tie Jun Cui

**Affiliations:** ^1^ State Key Laboratory of Millimeter WavesSoutheast UniversityNanjing210096P.R. China; ^2^ Synergetic Innovation Center of Wireless Communication TechnologyNanjing210096P.R. China; ^3^ School of Physical Science and TechnologySouthwest UniversityChongqing400715P.R. China; ^4^ Department of Electrical and Computer EngineeringNational University of SingaporeSingapore119620Singapore; ^5^ School of Physics and AstronomyUniversity of BirminghamEdgbastonBirminghamB15 2TTUK; ^6^ Cooperative Innovation Centre of Terahertz ScienceNo. 4, North Jianshe RoadChengdu610054P.R. China

**Keywords:** high‐resolution imaging, matched solid immersion lens, metamaterial, path shaping, transformation optics

## Abstract

**An all‐dielectric semispherical lens** with functions in shaping 3D wave‐propagation paths is proposed and experimentally verified. When radiation sources are placed in the central region, the lens behaves as a magnifying device to resolve the sources in subwavelength scale; while when the electromagnetic waves impinge on the semispherical lens from outside, they will be guided spirally inward.

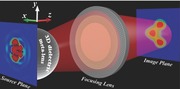

Transformation optics (TO) and metamaterials provide a novel strategy to direct and control the electromagnetic (EM) fields,^1^ based on which many exciting devices have been designed. As a typical application of metamaterials, subwavelength imaging lenses have attracted great attention. Breaking the diffraction limit of optical imaging resolution has always been the goal of engineers involved in the design of optical lens. The perfect lens based on ideal negative‐refraction metamaterials has been theoretically proposed to achieve perfect imaging by amplifying evanescent waves.^2^, ^3^ In this regard, superlens was experimentally demonstrated using a thin slab of silver or SiC.^4^, ^5^ To achieve the subdiffraction imaging in the far‐field region more efficiently, hyperlenses have been proposed using anisotropic materials with hyperbolic dispersions.^6^, ^7^, ^8^ Soon after, cylindrical hyperlens^9^, ^10^ and spherical hyperlens^11^ were experimentally demonstrated by radially stacking multilayers of metal–dielectric materials. In fact, both superlens and hyperlens can be precisely designed based on TO,^12^ however they are still facing obstacles pertaining to intrinsic absorption and narrowband properties. To avoid the limitation, a theoretical proposal of 2D magnified lens without negative‐refraction indices has been introduced and analyzed,^13^ and the experimental verification was conducted based on 2D nonresonant metamaterials.^14^

In order to achieve more promising and advanced control of EM waves using nonresonant metamaterials, we herein propose and validate a 3D broadband all‐dielectric metalens, which can shape the wave trajectory in 3D. In particular, the proposed semispherical lens made of isotropic and inhomogeneous dielectrics possesses high‐resolution imaging and facilitates omni‐directional absorptions simultaneously. When the radiation sources are placed in the lens' central region, the lens behaves as a magnifying device, a matched solid immersion lens (SIL) in GHz frequencies, to resolve the sources in subwavelength scale, while it enables an absorbing sphere to absorb incoming plane waves in all directions. The conventional SIL usually employs constant high‐index materials in optics. The principle of such optical SIL, however, has not been addressed in microwaves for which super‐resolution is also an important subject of study. Moreover, the direct translation from optics to microwaves will lead to critical index‐mismatch issue at the boundary, resulting in low operating efficiency in electromagnetic waves. Hence one function of our proposed SIL‐like device can cure this long‐lasting problem.

To verify the high‐resolution magnifying effects experimentally, we additionally design a 3D focusing lens using metamaterials based on geometrical optics. We experimentally demonstrate the first 3D broadband and low‐loss subwavelength imaging system for the far‐field subdiffraction resolution in the microwave frequencies. Benefiting from the low‐loss and broadband properties of the all‐dielectric metamaterial, the experimental results show excellent high‐resolution performance from 8 to 12 GHz. The metalens is capable of magnifying the subwavelength space between two objects, and the focusing lens subsequently produces a magnified image in the far‐field region, where the two objects are easily distinguished. To demonstrate the omni‐directional absorption facilitated by the same lens, we design a planar absorber located beneath the lens, and launch the incident waves at all spatial angles (from 0° to 90°) with respect to the normal line of the planar absorber. Experimental results show great improvement in enabling almost all‐angle absorption by using this metalens to physically “curve” the wave trajectory inside.

## Experimental Section

*Derivation of Matched SIL*: The experiment was started with a strict transformation‐optics design of the matched gradient‐refractive‐index metalens, in which a twofold transformation needed to be carried out. The real and virtual spaces are denoted by r, θ, φ and (r′, θ′, φ′), respectively. First, a spherical region (r′≤b−δ) in the virtual space was compressed into region I (r≤a) in the real space. Second, an annular region (b−δ<r′≤b) in the virtual space was stretched into region II (a<r≤b) in the real space. Then the far‐field pattern of two closely packed sources (*s*_1_ and *s*_2_) in the real space would be equivalent to that of two well‐separated sources (*s′*_1_ and *s′*_2_) in the virtual space, realizing the functionality of high‐resolution metalens, as illustrated in Figure S1 (Supporting Information).

Using the transformation optics procedure, the closed‐form expressions of material parameters for the metalens were obtained (see the Supporting Information). In the case of δ→0, the required refractive index of the 3D magnifying metalens was derived as

(1) $$n(r)=\{\begin{array}{cc} \frac{b}{a} & r\leq a\\ \mathrm{diag}\left(0,\;\;\;\frac{b}{r},\;\;\;\frac{b}{r}\right) & a<r\leq b \end{array}$$

It was found that the material of region II was anisotropic and the radial refractive index was close to zero. However, such an anisotropic material was very difficult to realize in wideband experimentally, and it was needed to simplify the material parameters. Numerical results suggested that the far‐field pattern was nearly invariant with the change of the radial refractive index. Thus, the following material property$n_{r}=n_{\varphi }=n_{\theta }=b/r$ was ingeniously selected and finally an isotropic metalens was obtained as

(2) $$n(r)=\{\begin{array}{c} b/a\;\;\;\;r\leq a\\ b/r\;\;\;a<r\leq b \end{array}$$

**Figure 1:**
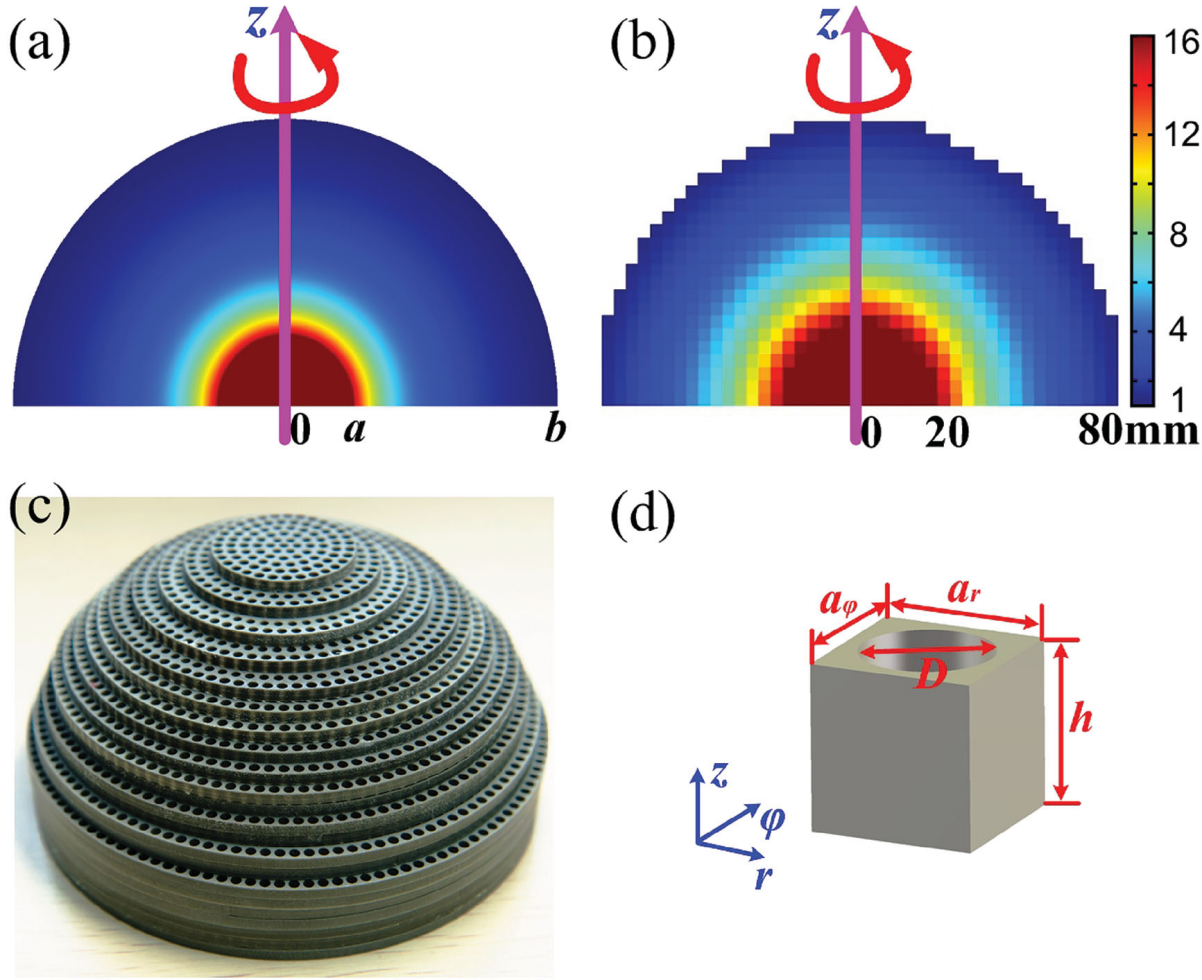
The transformation‐optics high‐resolution metalens. a) The relative permittivity distribution of the ideal high‐resolution metalens. b) The relative permittivity distribution of really fabricated high‐resolution metalens. c) The fabricated sample of high‐resolution metalens.d) The structured unit cell of the fabricated metalens.

It was obviously seen that the metalens was composed of a core with high refractive index and an impedance‐matching layer with the refractive index changing from that of the core to 1 for the free space, as shown in **Figure** **1**a. To understand the magnifying effect of the lens physically, the dispersion relation of time‐harmonic waves in an isotropic medium was studied

$$k_{x}^{2}+k_{y}^{2}+k_{z}^{2}=\frac{\omega^{2}n}{c^{2}}$$

in which *ω* is the radian frequency of the EM wave and *c* is the light velocity in free space. When the frequency *ω* was fixed, the equi‐frequency surface to be a sphere with radius *r = ωn* ^1/2^ */c* could be obtained. It was obvious that a bigger radius would generate larger amount of wave numbers for the imaging, and the radius was dependent on the refractive index. The function of the matching layer was to propagate the waves with magnified wave numbers smoothly to the free space without any loss.

*Choice of Unit Structures*: The metalens material parameters are nonmagnetic, isotropic, and inhomogeneous, which can be realized by nonresonant graded metamaterials. The metalens was fabricated using multilayered dielectric plates by drilling air holes of different spatial sizes to achieve the gradient distribution of refractive index (Figure 1a), as described in Equation (2). The dielectric permittivity distribution of the really fabricated metalens is illustrated in Figure 1b.

Figure 1c illustrates the schematic and fabricated sample of metalens. To obtain the required dielectric constants, first TiO_2_ and polyphenylene oxide were mixed with different concentration ratios to generate dielectric materials with dielectric constant between 3 and 16. On the other hand, the permittivity between 1 and 3 was achieved by two kinds of Teflon, one of which has the relative permittivity of 3 with loss tangent 0.0025, and the other has permittivity of 2.2 with loss tangent 0.001. The metalens was designed to work in broadband from 8 to 12 GHz, and choose four kinds of different‐material unit cells as the building blocks of metamaterials to realize the required distribution of refractive index. The size of each unit cell was *a_r_* × *a_ϕ_* × *h* mm^3^, as shown in Figure 1d. The above mentioned four kinds of plates with 3 mm thickness were chosen to generate all unit cells.

The gradient variation of refractive index (*n*) could be obtained by changing the diameters of air holes in the unit cells. The effective indices of refraction for such unit cells had been retrieved using the effective medium theory and S‐parameter retrieval method.^15^, ^16^ The weakly anisotropic property of unit cell has been studied in ref. 17 for 3D case by considering three orthogonal polarizations of incident waves. The equivalence between effective medium parameters and drilling‐hole unit cells has been verified in refs. 14 and 17 . In the fabrication, a semispherical metalens was manipulated and employed because the object to be imaged could be attached to the core of the lens. The fabricated sample was constructed by 22 layers of different dielectric‐plates, in which each layer was composed of the unit cells with subwavelength air holes in the center. The whole metalens was achieved by attaching the 22 layers together, including more than 20 000 unit cells, as displayed in Figure 1b. The details of air holes are illustrated in Table S1 in the Supporting Information.

**Figure 2:**
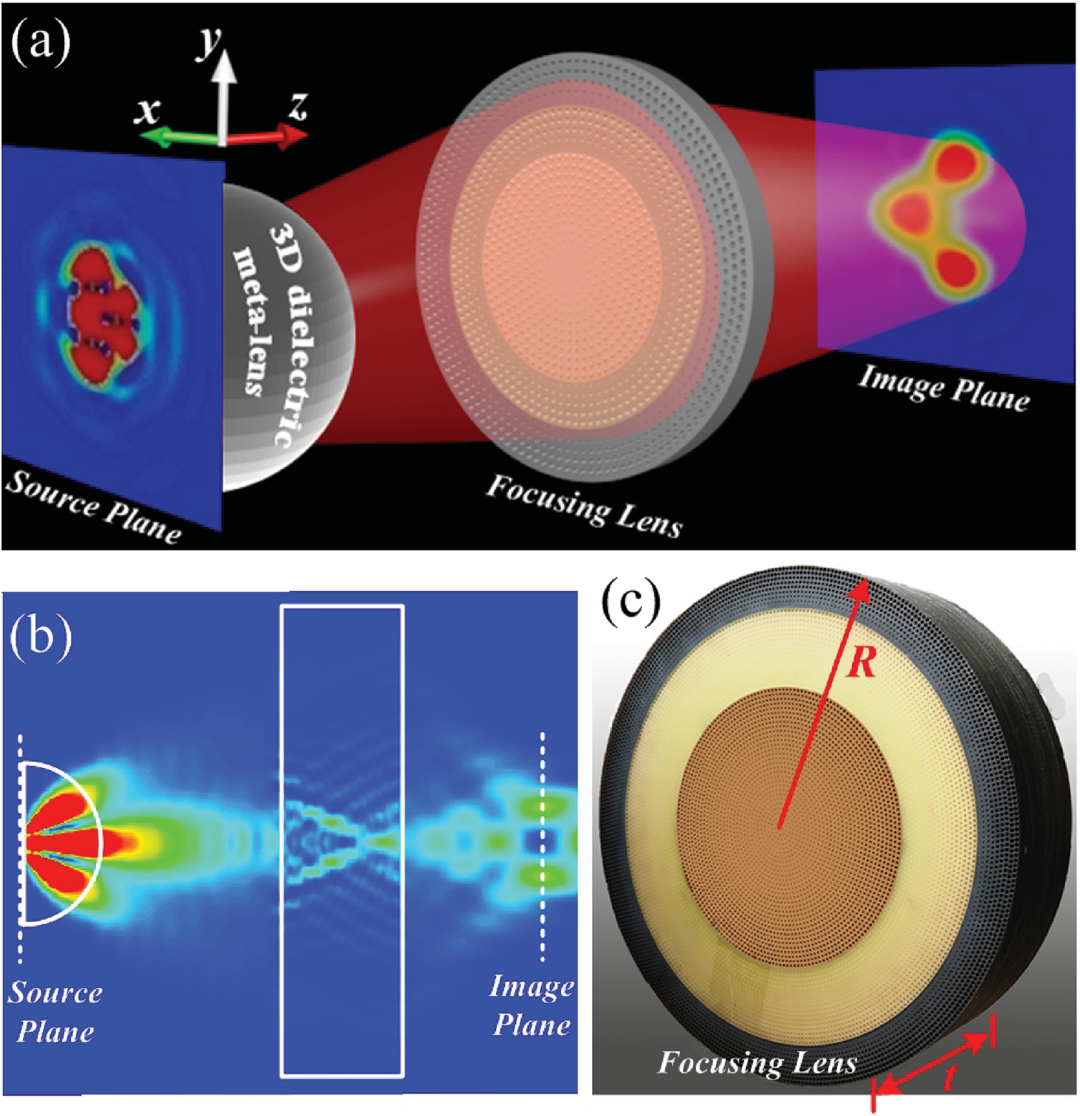
The experimental setup and full‐wave simulations of 3D metalens. a) The schematic illustration of the experimental setup for the magnifying metalens with the simulated E‐field distribution on the plane of three sources and E‐field distribution in the image plane. We use three small‐sized dipoles as the feeding sources to be magnified. The reverse image of the small‐sized dipole sources is clearly observed after four‐times magnification. b) The E‐field distribution in the incident plane. c) To display the magnifying performance of 3D metalens, a focusing lens is fabricated.

*Visualization of Experimental Results*: To visualize the high‐resolution imaging performance, a planar gradient‐index focusing lens was designed so as to form the far‐field subwavelength image of two active sources placed closely to each other. It was assumed that the planar lens was rotationally symmetric with thickness *t*, and the material parameters were varied along the *R* direction and were invariant along the *ϕ* and *z* directions. The principle of the planar gradient‐index lens has been demonstrated in Figure S1c (Supporting Information). In order to design the planar focusing lens, the spherical waves excited by a point source should be focused on one spot at the other side when propagating through the gradient‐index lens. The focusing distance is *f*_1_ away from the lens. According to the geometrical optics, if the source position and focusing spot are fixed, the refractive index *n* is obtained as

(3) $$n(R)=n_{c}-\frac{2\left(\sqrt{R^{2}+f^{2}}-f\right)}{t}$$

in which *n*_c_ is an arbitrary positive number, which denotes the refractive index at the center of planar lens. A judicious and practical choice is to make the refraction index of the lens easily accessible. Evidently, *n*(*R*) was only varied along the *R* direction. The focusing lens with radius *R* = 171 mm and the refractive index *n*_c_ = 2.6 were fabricated, as shown in **Figure** **2**c.

The principle of the 3D imaging system is illustrated in Figure 2a. There are two metamaterial‐based lenses in this system: a 3D semispherical high‐resolution dielectric metalens designed by TO and a planar focusing lens designed by geometric optics. The metalens will magnify the signal of the objects, which are located at the core of the semispherical lens. The focusing lens will then gather the magnified signal of the original objects and focus them to generate the image of the objects. Both lenses are isotropic and rotationally symmetric.

To evaluate the imaging performance of the metamaterials system, a 3D near‐field scanning system was employed to measure the electric‐field distributions in a horizontal plane at the level of the center of the magnifying metalens. The magnifying and focusing lenses were fixed on a rigid foam plate. A monopole probe was mounted in the space to detect the electric‐field distribution. In order to generate two feeding sources, the feeding signal was divided into two paths by using a power divider. Following the numerical simulations, the distance of two small‐distance sources was 7.5 mm, and the distance of two large‐distance sources was 30 mm. Such two feeding sources were located at the center of magnifying metalens. A moving stage was used to carry the metallic plate and scans in the *x* and *y* directions, so that the near‐field distributions in a certain area could be measured. The step resolution in both scanning directions was set as 1 mm.

**Figure 3:**
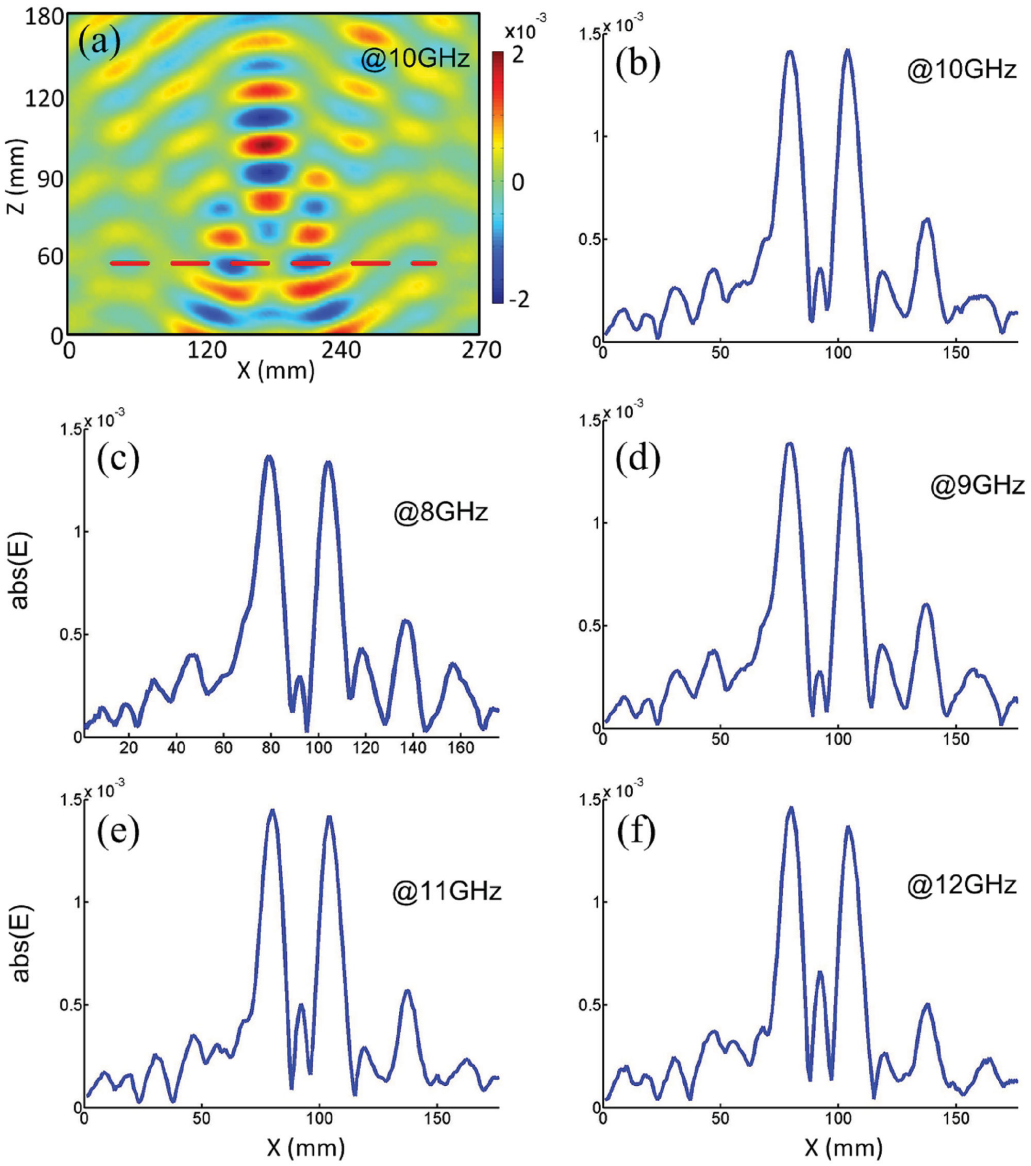
The experimental results of the high‐resolution dielectric metalens. a) The near‐field distribution of the high‐resolution metalens in the measurement region at the central frequency 10 GHz. The red dashed line denotes the image plane. The field distributions along the focusing line at b) 10 GHz, c) 8 GHz, d) 9 GHz, e) 11 GHz, and f) 12 GHz.

To validate the suggested 3D matched magnifying metalens experimentally, The fabricated sample (with geometrical parameters, *a* = 20 mm, *b* = 80 mm, and *d* = 7.5 mm) was measured in broadband from 8 to 12 GHz. The electric‐field distributions were scanned along a horizontal plane at the level of the center of the magnifying metalens. The measured near fields were plotted at 10 GHz in **Figure** **3**a. The red dashed line denotes the focusing plane, which serves as the observation line. To illustrate the imaging effect of the magnifying metalens, the field intensity was plotted along the observation line at 10, 8, 9, 11, and 12 GHz in Figure 3b–f. Note that the observation lines will be different at different frequencies. In all figures, it was observed that the two peaks, denoting images of two sources, can be evidently distinguished. It could be seen that the magnifying performance of the 3D metalens was very attractive due to its operation in a broad frequency band. Therefore, the high‐resolution and broadband properties were reached simultaneously using the proposed metalens.

To further verify the imaging lens, 3D full‐wave simulations were performed using commercial software, CST Microwave Studio. All geometrical sizes of the magnifying metalens and focusing lens were the same as those in experiments, and hence the designed magnification ratio was 4. To better illustrate the performance of the magnifying metalens, three 1/6‐wavelength‐long dipoles were designed as the feeding sources, which were attached on the inner surface. The separation among three dipoles was 7.5 mm. The near‐field distribution on the incident plane is shown in Figure 2b, which illustrates the EM waves from the sources to the imaging plane. As shown in Figure 2b, at the imaging plane, there are images of only two sources displayed but the image of the other source does not appear because the distances among the real sources have been magnified by the high‐resolution metalens, which falls out of the cut plane that was selected to plot the field. Figure 2a shows the near‐field distributions of three dipoles, which are difficult to distinguish. The electric‐field distributions on the focusing plane, however, clearly show how the three images of the diploes were magnified to three bright spots, as shown in Figure 2a, with the separation of 30 mm, which were in good agreements with the analytical calculations, showing a magnification of 4 in both *x* and *y* directions. By increasing the ratio between the inner and outer radii of the magnifying metalens, the magnification and resolution can be furtherimproved.

**Figure 4:**
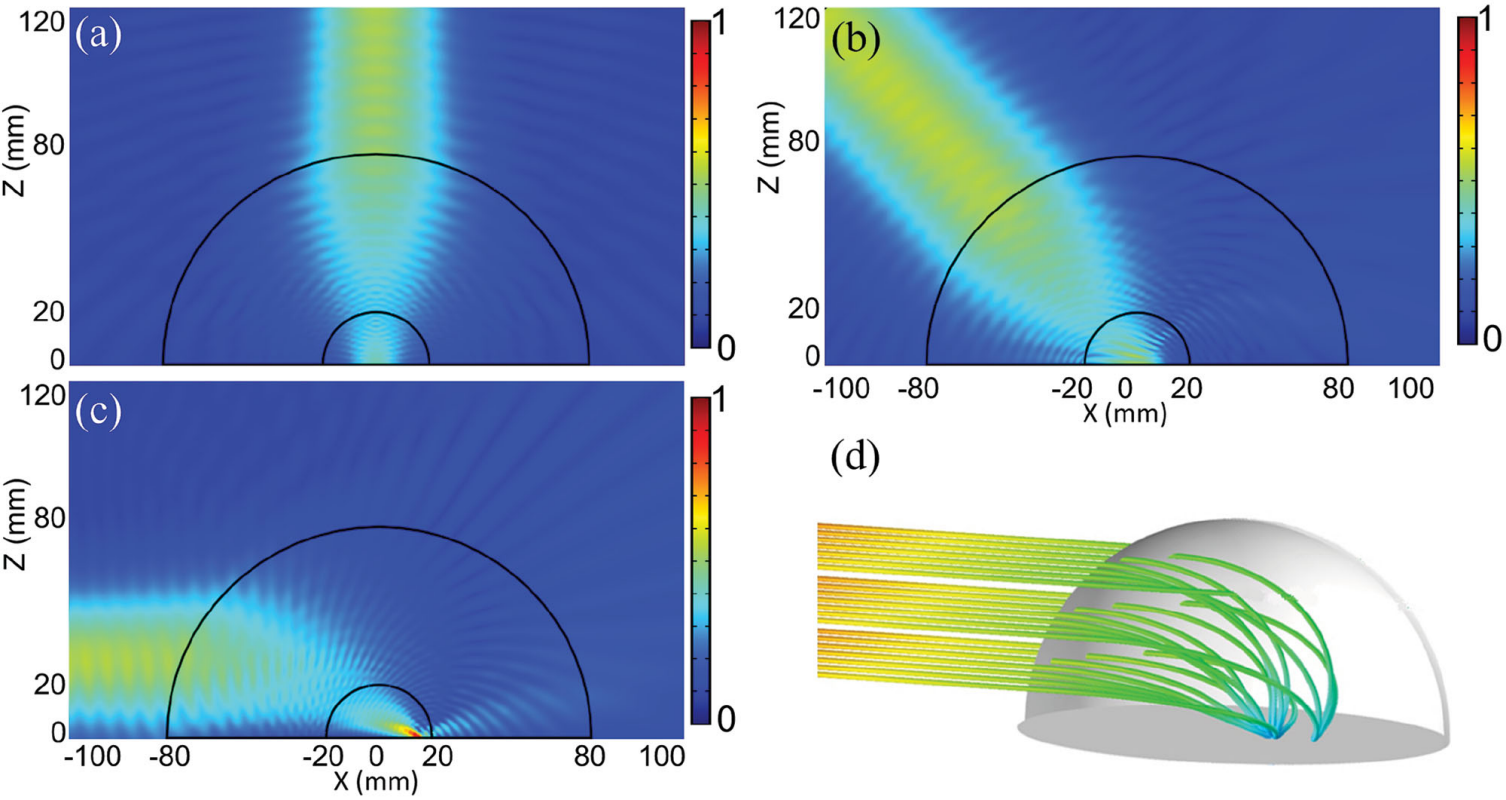
The results of wave‐path shaping performance of the metalens. a) The simulated E‐field amplitude distribution when a Gaussian‐beam is incident along the optical axis. b) The simulated E‐field amplitude distribution when a Gaussian‐beam is incident at an oblique angle of 45°. c) The simulated E‐field amplitude distribution when a Gaussian‐beam is incident horizontally. d) The ray‐tracing result of the metalens when the waves are incident horizontally.

*Wave‐Path Shaping Performance*: It was noted that the high‐resolution metalens was spherically symmetric with the radial variation of permittivity and the index matching on the corresponding interfaces. Hence, it was believed that when the waves are incident from outside onto the graded lens, they will intrude into the lens and propagate without any reflections. To demonstrate the wave‐path shaping performance of the gradient‐index lens in microwave frequencies, the incidence of Gaussian beam was considered. **Figure** **4**a,b illustrates the amplitude distributions of simulated electric fields Abs(*E*_z_) at 10 GHz when a Gaussian beam was incident on the semispherical lens along the optical axis and at an oblique angle of 45° tilted from the optical axis, respectively. It was noted that all on‐axis waves were directly attracted by the lens without reflections, and nearly all off‐axis rays bended in the gradient‐index region spirally and then were trapped to the core. It was clear that the incident beam became convergent inside the shell region and then entered the lens core, instead of being divergent in the free‐space radiations.

An extreme case was considered. When the beam was incident to the lens along the horizontal direction, all the rays were bent toward the lens' central region and travelled around the shell spirally. The full‐wave simulated field distribution is illustrated in Figure 4c, and the ray‐tracing result is shown in Figure 4d. Both the full‐wave and ray‐tracing results illustrate that nearly all incident waves impinged on the lens could be curved and then trapped at the center. In order to quantitatively illustrate such effect, an experimental measurement was performed. It was observed that the transmission coefficient decreased by 15 dB, from 9.9 to 10.5 GHz, with the wave‐path shaping lens compared to the case without the metalens. The detail is shown in the Supporting Information.

SIL is important for imaging, data storage, photolithography, and spectroscopy.^18^, ^19^ Due to impetance mismatched with free space, the transfer efficiency of SIL is relatively low. A 3D broadband matched SIL lens based on gradient‐refractive‐index metamaterials was proposed and constructed. It can enlarge the distance of two neighboring sources and obtain a far‐field high‐resolution image with the assistance of a planar focusing lens. On the other hand, when the waves are incident on the metalens from outside, they will be guided and trapped into the center of the metalens. As a subapplication, the metalens can also be used to reduce the size of the radiators. Such lenses were fabricated in the microwave frequency using all‐dielectric metamaterials. The experimental results have verified the image‐magnification and wave‐trapping performance of the dielectric metalens in GHz frequency. Owing to the low loss and all‐dielectric properties, the proposed lens system can be used for far‐field imaging with high resolution and electromagnetic energy collections.

## Supporting information

As a service to our authors and readers, this journal provides supporting information supplied by the authors. Such materials are peer reviewed and may be re‐organized for online delivery, but are not copy‐edited or typeset. Technical support issues arising from supporting information (other than missing files) should be addressed to the authors.

Supplementary
