# Supplementary material for: Shaping 3D Path of Electromagnetic Waves Using Gradient‐Refractive‐Index Metamaterials
Source: Adv Sci (Weinh). 2016 Mar 15;3(8):1600022. doi: 10.1002/advs.201600022 (PMC5115487; doi:10.1002/advs.201600022)
Supplement: Supplementary file 1 — Supplementary [file ADVS-3-0k-s001.pdf]

Copyright WILEY-VCH Verlag GmbH & Co. KGaA, 69469 Weinheim, Germany, 2016.

ADVANCED SCIENCE
Open Access

Supporting Information

for *Adv. Sci.*, DOI: 10.1002/advs.201600022

Shaping 3D Path of Electromagnetic Waves Using Gradient-Refractive-Index Metamaterials

Wei Xiang Jiang, Shuo Ge, Tiancheng Han, Shuang Zhang, Muhammad Qasim Mehmood, Cheng-Wei Qiu, and Tie Jun Cui**

Supporting Information

Shaping electromagnetic wave paths using three-dimensional gradient-refractive-index metamaterials

Wei Xiang Jiang, Shuo Ge, Tiancheng Han, Shuang Zhang, Muhammad Qasim Mehmood, Cheng-Wei Qiu, and Tie Jun Cui**

This Supporting Information includes some details of the designing high-resolution meta-lens, focusing lens, planar absorber and more test results.

1. Derivation of the transformation-optics high-resolution meta-lens

To compress the spherical region $(r' \le b-\delta)$ in the virtual space into Region I $(r \le a)$ in the real space, the following transformation is used

$$r = \frac{a}{b-\delta} r', \ \phi = \phi', \ \theta = \theta'. \tag{1}$$

Then the relative constitutive parameters for the core region $(r \le a)$ are expressed as

$$\varepsilon = u = diag\left(\frac{b-\delta}{a}, \frac{b-\delta}{a}, \frac{b-\delta}{a} \right) \tag{2}$$

To stretch the annular region $(b-\delta < r' \le b)$ in the virtual space into Region II $(a < r \le b)$ in the real space, we apply for the following transformation

$$r = \frac{b-a}{\delta}(r'-b)+b, \ \phi = \phi', \ \theta = \theta', \tag{3}$$

and the relative constitutive parameters for Region II are calculated as

$$\varepsilon = u = diag\left(\frac{b-a}{\delta}\left(\frac{r'}{r}\right)^2, \frac{\delta}{b-a}, \frac{\delta}{b-a} \right) \tag{4}$$

Eqs. (2) and (4) can be replaced by the refractive index and simplified as

$$n(r)=\begin{cases}\dfrac{b-\delta}{a} & r\le a\\ diag\left(\dfrac{\delta}{b-a},\dfrac{r'}{r},\dfrac{r'}{r}\right) & a<r\le b\end{cases}. \tag{5}$$

When $\delta\to 0$, Eq. (5) will be reduced to Eq. (1) in the main text.

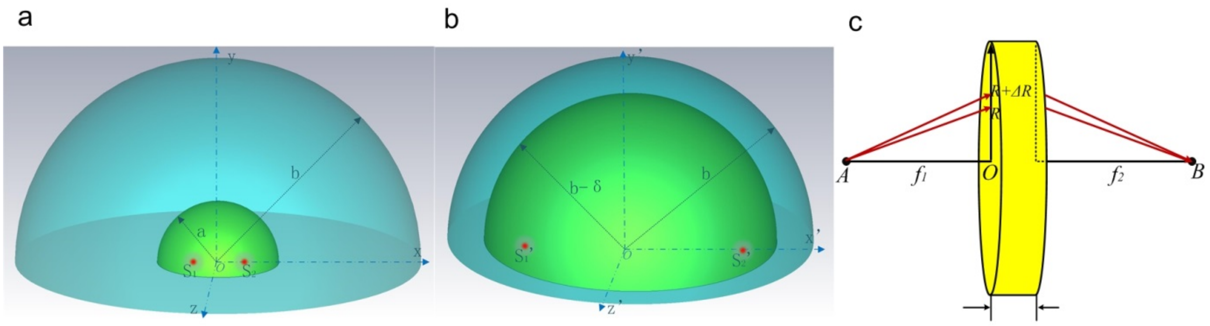

Figure S1. Illustrations of the high-resolution meta-lens and planar focusing lens. (a) Physical space for the design of high-resolution meta-lens. (b) Virtual space for the design of high-resolution meta-lens. (c) The principle of planar focusing lens.

2. Simulation results of 3D high-resolution meta-lens

We first validate the performance of 3D high-resolution meta-lens numerically. We select the geometrical parameters a=20 mm, b=80 mm, the operating frequency f_0=10GHz, and the distance between two line sources d=7.5 mm. We assume that the high-resolution meta-lens is nonmagnetic (μ=1) in simulations and experiments, and the relative permittivity distribution is shown in Fig. 1(c). Note that the distance of two sources is about 0.25 wavelengths, which is far below the Abble diffraction limit. To verify both the near-field and far-field effects of the fabricated high-resolution meta-lens, we compute the near-field distributions and radiation patterns, respectively. Figures S2(**a**) and (**b**) show the simulation results of two small-distance sources (the distance between them is d) with and without the 3D high-resolution meta-lens, respectively. The left panel of Fig.S2**a** illustrates the near-field distribution, which is similar to that of a single monopole antenna in two dimensions due to the deep subwavelength separation between the two sources.

As we see in the middle panel of Fig.S2**a**, the far-field distributions on the x-y plane is nearly homogeneous, behaving as a monopole. In such a case, two sources are very difficult to be distinguished, i.e., they look like a single source in the far-field region. To distinguish the two sources, we put the small-distance sources clinging to the core of the 3D semi-spherical meta-lens. We present the near-field distributions of the two sources within the

high-resolution meta-lens in the left panel of Fig.S2**b**, which is remarkably different from that of single source. In the far fields, the radiation pattern changes significantly. In the left panel of Fig.S2**c**, we illustrate the near-field distribution of two sources with large distance *d* in the free space without the high-resolution meta-lens, which shows very similar features as the left panel of Fig. S2**b**. We also plot the far-field radiation pattern in the right panel of Fig.S2**c**, which is almost identical to the right panel of Fig. S2**b**. Hence, with the proposed high-resolution meta-lens, two sources with a deep subwavelength separation can be easily differentiated beyond the stipulated diffraction limit. We observe that the subwavelength-distance sources inside the high-resolution meta-lens have equivalent far-field signatures to the large-distance sources in the free space.

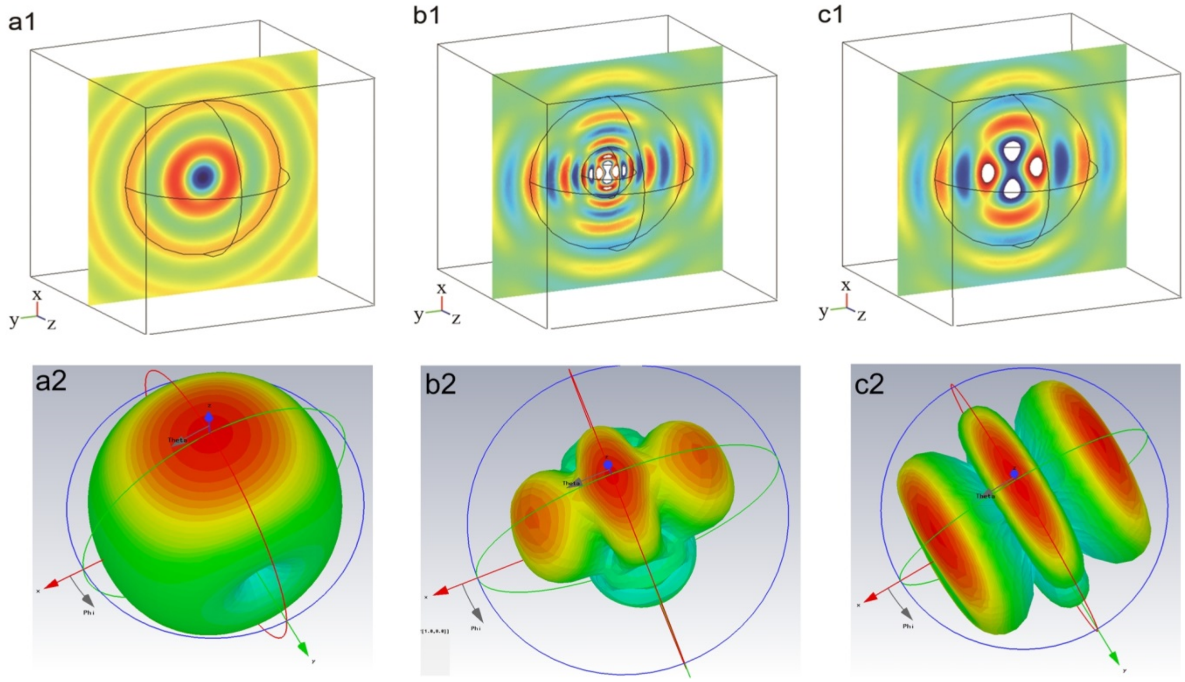

Figure S2. The simulation results of the designed 3D high-resolution meta-lens at 10GHz. (a) The near-field distribution of two sources with small distance (7.5mm) in free space (upper row), and the far-field pattern (lower row). (b) The near-field distribution of two sources with small distance (7.5mm) inside the meta-lens (upper row), and the far-field pattern (lower row). (c) The near-field distribution of two sources with large distance (22.5mm) in free space (upper row), and the far-field pattern (lower row).

3. The design parameters of the high-resolution meta-lens and focusing lens

The design parameters of the high-resolution meta-lens and planar focusing lens are presented in Figs. S3-S4, and Table S1.

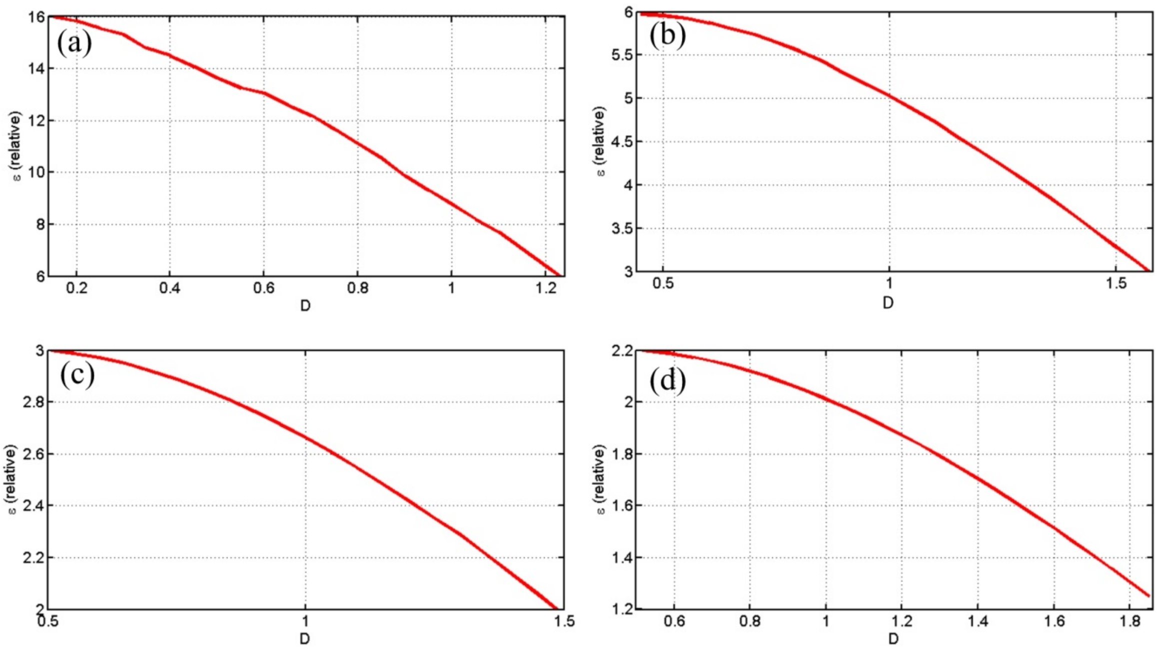

Figure S3. The retrieval material parameters of unit cell for high-resolution meta-lens. (a) Material 1: TiO_2-polyphenylene oxide mixer with the dielectric constant 16. (b) Material 2: TiO_2-polyphenylene oxide mixer with the dielectric constant 6. (c) Material 3: Teflon with the dielectric constant 3. (d) Material 4: Teflon with the dielectric constant 2.2.

Table S1. Details of the air-hole radii in 3D high-resolution meta-lens. The red numbers denote the radii of air holes in TiO2-polyphenylene oxide mixer with the dielectric constant 16, the green numbers denote the radii of air holes in TiO2-polyphenylene oxide mixer with the dielectric constant 6, the blue numbers denote the radii of air holes in Teflon with the dielectric constant 3, and the black numbers denote the radii of air holes in another Teflon with the dielectric constant 2.2.

Layer 1	Layer 2	Layer 3	Layer 4	Layer 5	Layer 6	Layer 7	Layer 8	Layer 9	Layer 10	Layer 11
0	0	0	0	0	0	0	0	0.8	1	1.15
0	0	0	0	0	0	0	0	0.85	1.05	1.15
0	0	0	0	0	0	0	0	0.9	1.05	1.2
0	0	0	0	0	0	0	0.75	0.95	1.1	1.2
0	0	0	0	0	0	0.65	0.9	1.05	1.15	0.2
0	0	0	0	0	0.65	0.85	1	1.1	1.2	0.45
0	0	0	0.5	0.7	0.85	1	1.1	1.2	0.3	0.6
0.65	0.7	0.75	0.85	0.9	1	1.1	1.2	0.2	0.55	0.75
0.9	0.95	0.95	1	1.1	1.15	1.2	0.25	0.55	0.75	0.9
1.1	1.1	1.1	1.15	1.2	0.15	0.35	0.6	0.75	0.9	1
1.2	1.2	0.15	0.15	0.35	0.55	0.65	0.8	0.9	1	1.1
0.5	0.5	0.55	0.6	0.7	0.75	0.85	0.95	1	1.1	1.15
0.75	0.75	0.8	0.85	0.9	0.95	1	1.05	1.1	0.15	0.5
0.95	0.95	0.95	1	1.05	1.05	1.1	1.15	0.35	0.55	0.75
1.1	1.1	1.1	1.1	1.15	0.15	0.35	0.55	0.65	0.8	0.9

0.25	0.25	0.3	0.4	0.5	0.6	0.7	0.8	0.85	0.95	0.45
0.65	0.65	0.7	0.7	0.75	0.85	0.9	0.95	0.4	0.6	0.75
0.9	0.9	0.9	0.95	0.15	0.35	0.5	0.6	0.75	0.85	0.95
0.5	0.5	0.55	0.6	0.65	0.7	0.8	0.85	0.95	1.05	1.1
0.8	0.8	0.85	0.85	0.9	0.95	1	1.05	1.1	1.2	1.25
1	1	1.05	1.05	1.1	1.1	1.15	1.2	1.25		
1.2	1.2	1.2	1.2	1.25	1.25	1.3				

Layer 12	Layer 13	Layer 14	Layer 15	Layer 16	Layer 17	Layer 18	Layer 19	Layer 20	Layer 21	Layer 22
0.25	0.65	0.85	1	1.15	0.5	0.8	0.2	0.65	0.9	1.1
0.3	0.65	0.9	1.05	1.15	0.5	0.8	0.25	0.7	0.95	1.1
0.4	0.7	0.9	1.05	1.15	0.55	0.8	0.3	0.7	0.95	1.15
0.5	0.75	0.95	1.05	0.15	0.6	0.85	0.4	0.75	1	1.15
0.6	0.85	1	1.1	0.35	0.7	0.9	0.5	0.8	1	1.2
0.7	0.9	1.05	1.15	0.5	0.75	0.95	0.6	0.85	1.05	1.2
0.8	0.95	1.1	0.2	0.6	0.85	0.35	0.7	0.95	1.1	
0.9	1.05	1.15	0.45	0.7	0.9	0.55	0.8	1	1.15	
1	1.1	0.25	0.6	0.85	0.3	0.65	0.9	1.05		
1.1	0.15	0.55	0.75	0.95	0.55	0.8	1	1.15		
1.15	0.45	0.7	0.9	0.4	0.7	0.9	1.1			
0.45	0.7	0.85	0.3	0.65	0.85	1	1.2			
0.7	0.85	0.2	0.6	0.8	1	1.1				
0.85	0.25	0.6	0.8	0.95	1.1	1.2				
0.35	0.6	0.8	0.95	1.1	1.2	1.35				
0.65	0.85	0.95	1.1	1.2						
0.9	1	1.1	1.2							
1.05	1.15	1.2								
1.2	1.25									

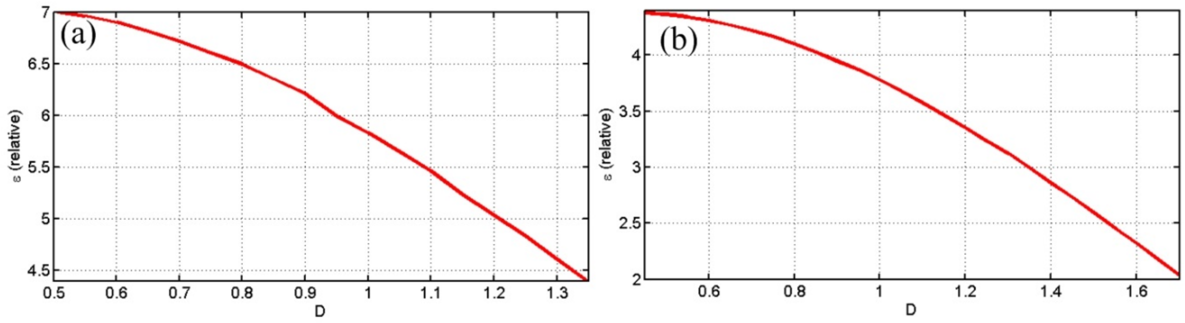

Figure S4. The retrieval material parameters of unit cell for the planar focusing lens. (a) Material 1: TP2 with the dielectric constant 7. (b) Material 2: FR4 with the dielectric constant 4.4.

4. Derivation of the ray-optics focusing lens

We assume that the planar focusing lens is rotationally symmetrical and the material parameters are varied along the R direction and invariant along the φ and z directions. The

principle of the planar gradient index lens has been demonstrated in Fig. 3(a). Consider two rays are incident on the planar lens with thickness t and enter the slab at locations R and $R+\Delta R$ along the slab surface, respectively. When the rays propagate through the slab, they will have different phase shifts. In another word, their optical path lengths will be different. In order to design the planar focusing lens, the spherical waves excited by a point source should be focused on one spot on the other side when propagating through the planar lens, as shown in Fig. 3(a). The point source is located on the position with the distance f_1 away from the planar lens and the focusing spot with the distance f_2 at the other side away from the lens. According to the geometrical optics, if the source position and focusing spot are fixed, the refractive index n can be obtained. Considering two arbitrary rays, as illustrated in the Fig. 3(a), the relationship between their optical paths should be equal:

$$\sqrt{f_1^2+R^2}+\sqrt{f_2^2+R^2}+n(R)t=\sqrt{(R+\Delta R)^2+f_2^2}+\sqrt{(R+\Delta R)^2+f_1^2}+n(R+\Delta R)t. \quad (8)$$

After some algebra, we have

$$\frac{n(R)-n(R+\Delta R)}{\Delta R}t=\frac{\sqrt{(R+\Delta R)^2+f_1^2}-\sqrt{R^2+f_1^2}}{\Delta R}+\frac{\sqrt{(R+\Delta R)^2+f_2^2}-\sqrt{R^2+f_2^2}}{\Delta R}. \quad (9)$$

When $\Delta R \rightarrow 0$, we get the following differential equation

$$-\frac{dn}{dR}t=\frac{R}{\sqrt{R^2+f_1^2}}+\frac{R}{\sqrt{R^2+f_2^2}}, \quad (10)$$

By solving the above equation, we have

$$n(R)=n_c-\frac{\sqrt{R^2+f_1^2}-f_1}{t}-\frac{\sqrt{R^2+f_2^2}-f_2}{t}, \quad (11)$$

in which n_c is an arbitrary positive number which denotes the refractive index at the center of the focusing lens. A wise and practical choice of n_c is the easy realization of the lens refractive index. Evidently, $n(R)$ is only varied along the R direction.

5. Experiment results of the focusing lens

To verify the focusing effect of the planar focusing lens, we measure near-field distributions of the focusing lens when the monopole source is put on the focusing point. The measured results are shown in Fig. S5.

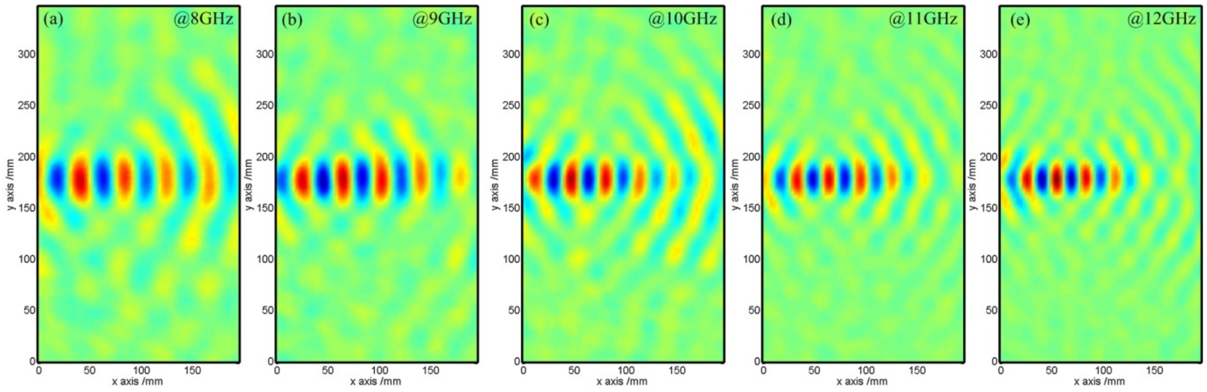

Figure S5. The normalized measured near-field distributions of the planar focusing lens.

6. Experiment results of the high-resolution meta-lens

We also measured the near-field distribution of the high-resolution meta-lens, and the results are shown in Fig. S6.

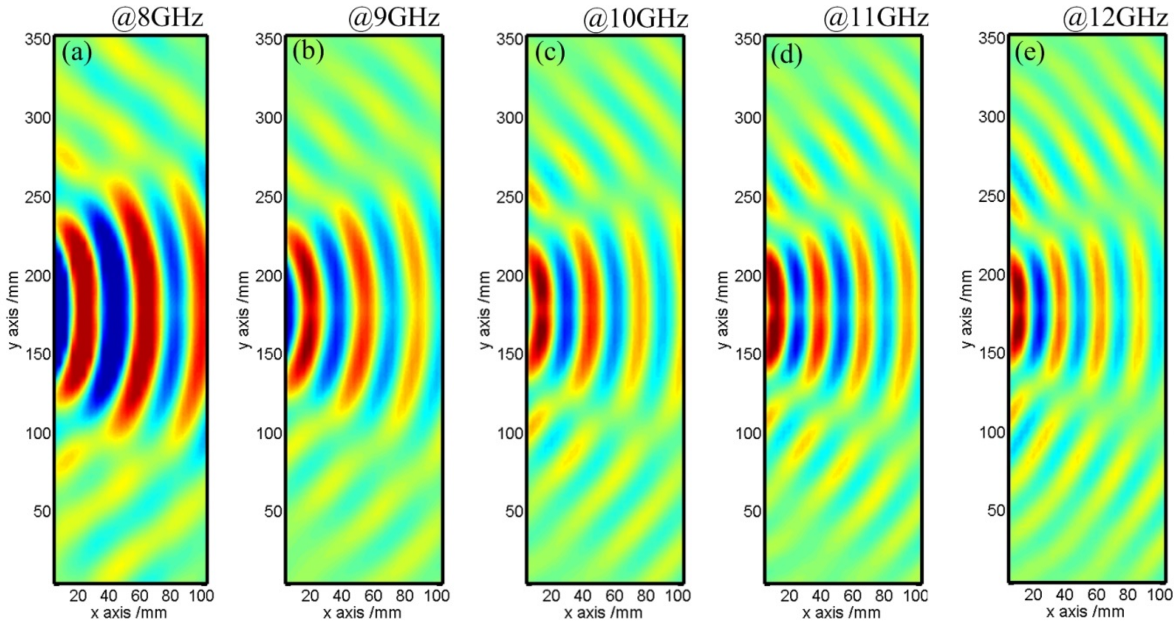

Figure S6. The normalized measured near-field distributions of the high-resolution meta-lens.

7. The designed and fabricated planar absorber

To demonstrate the wave-path shaping performance of the proposed dielectric meta-lens experimentally, we design and fabricate a planar absorber[1] to place under the 3D meta-lens. When the waves are guided to the bottom of the 3D meta-lens, the planar absorber will absorb

the waves in a high efficiency. The closed-ring structure is used as the basic unit cell [1]. The planar absorber shown in Fig. S7 is fabricated using copper-backed print circuit board (PCB) technology. To broaden the working band of planar absorber, we employ four closed-ring structures to form a new unit. The side length of adjacent sub-structures is increased by 1 mm.

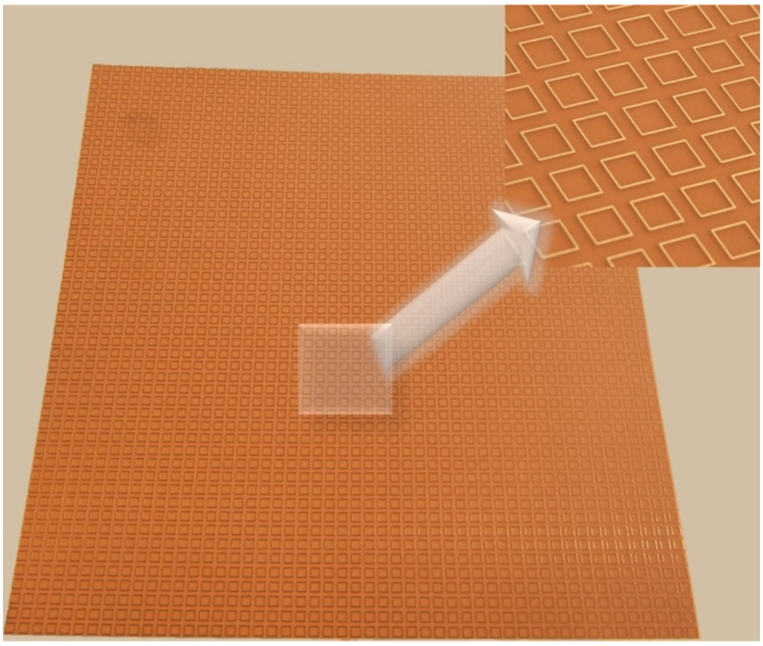

Figure S7. The fabricated planar absorber, in which the inset shows the details of unit cells.

8. The measurement of the beam-shaping performance of meta-lens

To demonstrate the extreme wave-guiding performance of such meta-lens experimentally, we perform a comparison measurement. First, we measured the transmission coefficient of a pair of horns aperture to aperture without the semi-sphere meta-lens and the planar absorber. After that, we measured the transmission coefficient of the pair of horns based on the experimental setup illustrated in Fig. S8(a), in which the transmitting antenna is put on the left and the receiving antenna on the right. Finally, we plot the net transmission coefficient, the transmission coefficient with the semi-sphere lens minus that without the semi-sphere lens. It is observed that the transmission coefficient is decreased by 15dB, from 9.9 to 10.5 GHz, with the wave-path shaping lens compared to the case without the meta-lens. Hence, nearly all incident waves impinged on the lens can be curved and then trapped at the center. We note that although the semi-sphere meta-lens can operate from 10 to 12 GHz, the planar absorber work only from 9.9 to 10.5 GHz.

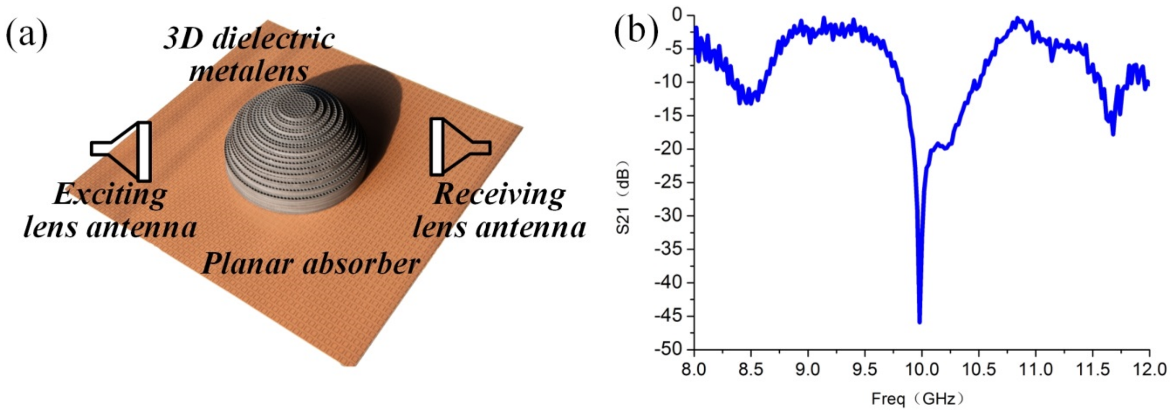

Figure S8. (a) The experimental setup to verify the wave-path shaping performance of the meta-lens. (b) To verify the wave-path shaping performance of the meta-lens in experiments, we plot the net transmission coefficient (the transmission coefficient with the semi-sphere lens minus that without the semi-sphere lens).

9. Addtional sub-application of meta-lens

Finally, we discuss additional sub-application of magnifying devices for the antenna[2-3]. As a sub-application of magnifying lens, it is used to reduce the size of the radiators. We made some simulations on the applications of the magnifying lens. The near-field distributions and far-field patterns are shown in the following figures. It is clearly observed that the directivity of the radiators, the line source and horn feeding, has been increased remarkably. We note that the radius of the center magnified region of our magnifying lens is 20 mm and the center working frequency is 10GHz, so the size of the radiator should be smaller than the magnified core. In the simulation, we set the size of the radiators (line source or a coaxial-to-waveguide coupler) as 8 mm, about a half size of center magnified region. The numerical results show that the directivities of the radiators are enhanced remarkably, which means the magnifying lens here can be also served for antenna application.

References

[1] Shen, X., Cui, T. J., Zhao, J., Ma, H. F., Jiang, W. X. and Li, H. Polarization-independent wide-angle triple-band metamaterial absorber. *Opt. Express* **2011**, 19, 9401.

[2] Luo, Y., Zhang, J., Chen, H., Huangfu, J. and Ran, L. High-directivity antenna with small antenna aperture. *Appl. Phys. Lett.* **2009**, 95, 193506.

[3] Tichit, P.-H., Burokur, S. N., and Lustrac, A. Reducing physical appearance of electromagnetic sources. *Opt. Express* **2013**, 21, 5053.

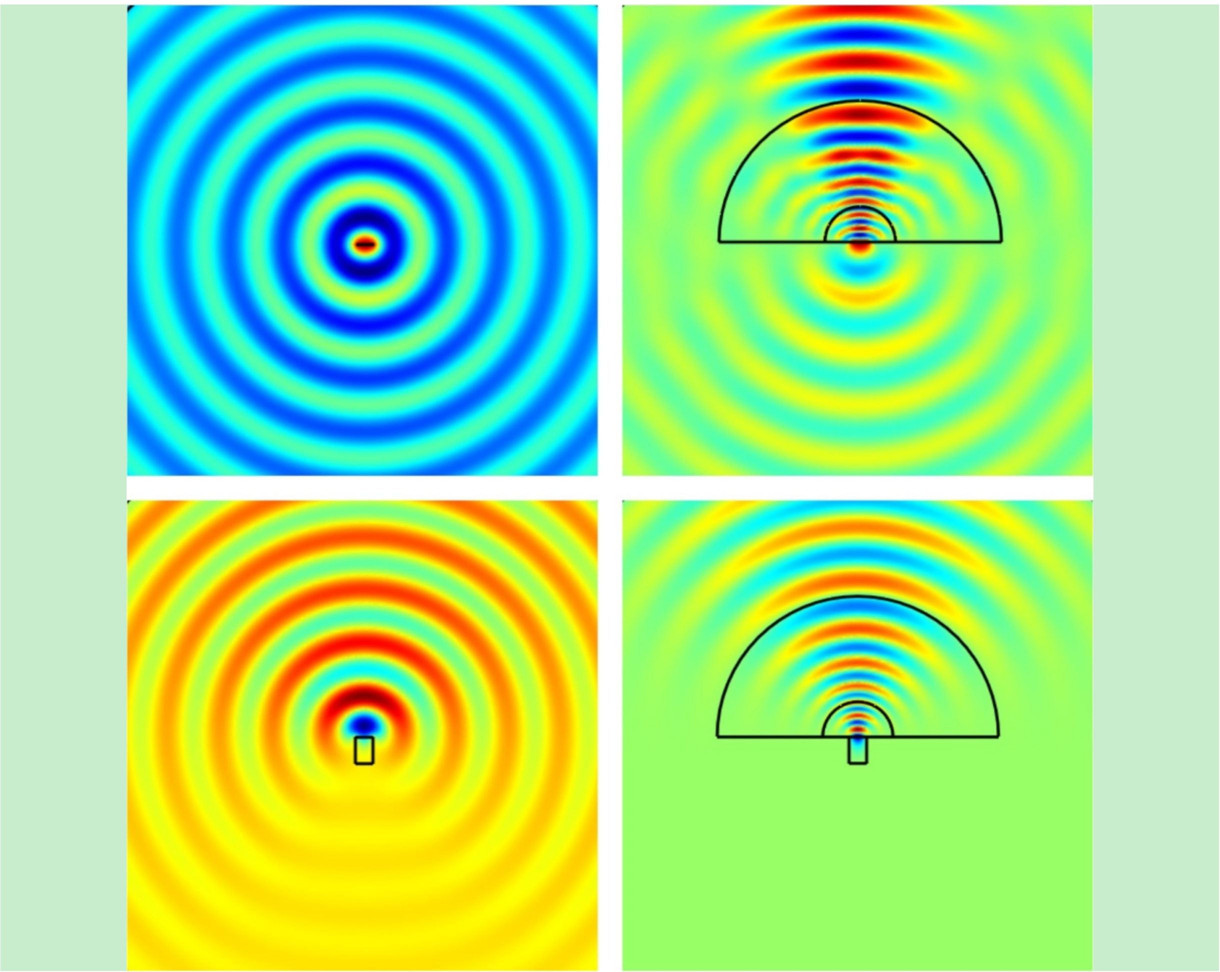

Figure S9. The cross-section near-field distribution of magnifying lens for antenna applications. Upper row: a line source without (left) and with (right) magnifying lens. Lower row: a coaxial-to-waveguide coupler feeding without (left) and with (right) magnifying lens.

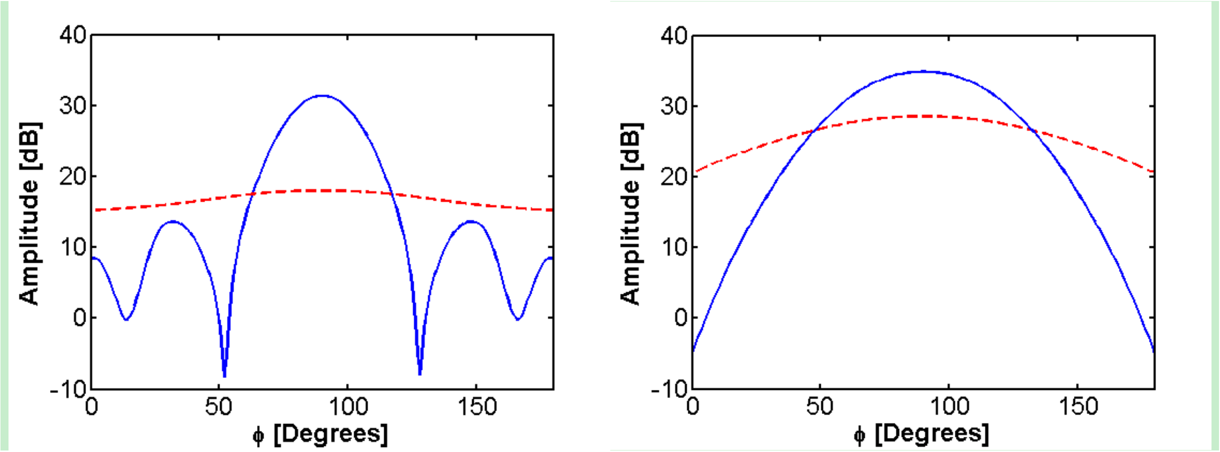

Figure S30. The far-field radiation patterns of the radiators with (blue solid line) and without (red dashed line) the magnifying lens. Left: a line source, Right: a coaxial-to-waveguide converter feeding.
